# Responsibility in Administering an Anesthetic

**Published:** 1898-09

**Authors:** 


AMERICAN JOURNAL

—OF—

DENTAL SCIENCE.

VOL. XXXII. BALTIMORE, SEPTEMBER, 1898. NO. 5.

### Selections.

ARTICLE I.

### RESPONSIBILITY IN ADMINISTERING AN ANESTHETIC.

A discussion has been carried on in a provincial lay contemporary upon the question, Who is responsible in practice for the conduct of the administration of an anesthetic? Several deaths occurred at a provincial hospital, and upon these the local press; it appears commented. The question of such reeponsibility is, however, one which has a much wider interest and importance. Usually the patient admitted into a hospital is received by a resident house surgeon or medical officer, who examines his condition and reports to his superior, the visiting surgeon. When an operation becomes imperative, the latter decides upon it, and an administrator of the anesthetic comes upon the scene. He may he a house surgeon, senior student, or a special officer, a member of the staff specially appointed to give all anesthetics. Who now decides upon what anesthetic is to be given, what method employed, and in extreme cases whether or not any anesthetic shall be given? A hospital surgeon of long standing and wide experience, in commenting upon the subject, contends that these questions lie solely within the province of the surgeon in charge of the case, upon the ground that he, and he only, has the opportunity of studying the case, and so is the best qualified to sit in judgment upon the points at issue. Upon the other hand, a gentleman in general practice writes that, at least as regards a private practice, he considers the surgeon as the least competent, since the family doctor, he alleges, knows all about the patient, while the surgeon views him wholly from the standpoint of his surgical malady. Probably much may be said on either side. Certainly in cases when special anesthetics are called in, the problem is more complex than when the surgeon calls upon a house surgeon to give chloroform, and also occasionally to assist him at the same time. If competent medical men, who devote themselves to the study of what is admittedly a large subject, and one not free from special difficulties and requiring some special training, are found willing to practice solely in the capacity of anesthetist—and that such is the case is shown in the past by the valuable work of such men as Snow and Clover—it becomes a grave question whether or not they should be regarded as mere experts in the methods of giving an anesthetic and accepting no responsibility beyond that involved in the technique of their calling, or whether, on the other hand, they have a right to determine what anesthetic is best in any given case under any given condition. It is urged on behalf of the anesthetists, with some show of reason, that when an expert is called upon to give the anesthetic, with him should rest the onus of selecting an appropriate anesthetic and the responsibility of giving it in such a manner as to impede as little as possible the manipulation of the surgeon, and afford the patient the greatest chance of getting safely through the operation. In all cases of special doubt or difficulty, a consultation between the administrator and the operator should enable the former to examine the patient and ascertain the surgeon's views and wishes.—*Lancet.*

